# The promotion of cervical cancer progression by signal transducer and activator of transcription 1-induced up-regulation of lncRNA MEOX2-AS1 as a competing endogenous RNA through miR-143-3p/VDAC1 pathway

**DOI:** 10.1080/21655979.2021.1947174

**Published:** 2021-07-05

**Authors:** Xiao-xing Liu, Qi-xiu Bao, Yan-mei Li, Yan-hua Zhang

**Affiliations:** a Department of Obstetrics and Gynecology, Dongying People’s Hospital, Dongying, Shandong, P.R. China; b Department of Public Health, Dongying People’s Hospital, Dongying, Shandong, P.R. China

**Keywords:** LncRNA MEOX2-AS1, STAT1, miR-143-3p, VDAC1, metastasis, biomarker

## Abstract

Long non-coding RNAs (lncRNAs) are the new regulators and biomarkers for various tumors. However, in cervical cancer (CC), the potential roles of lncRNAs are not well characterized. This research aimed at exploring the roles of MEOX2 antisense RNA 1(MEOX2-AS1) in CC progression and the underlying mechanisms. The examination of MEOX2-AS1 levels in CC specimens and cell lines was conducted by RT-PCR. Loss-of-function experiments were performed for the assays of proliferation, migration, and invasion of CC cells after various treatments. Animal experiments were applied for the determination of the effects of MEOX2-AS1 in vivo. Bioinformatics analysis, together with dual-luciferase reporter assays, was applied to demonstrate the possible relationships among MEOX2-AS1, miR-143-3p and VDAC1. In the paper, we reported that MEOX2-AS1 levels were distinctly upregulated in CC cells and tissues, and higher MEOX2-AS1 expressions indicated a poor clinical outcome. Besides, STAT1 could activate transcriptions of MEOX2-AS1 by binding directly to its promoter region. The silence of MEOX2-AS1 suppressed the metastatic and proliferative ability of CC cells, as revealed by functional assays. Mechanistically, MEOX2-AS1 sponged miR-143-3p to regulate VDAC1 expressions. Furthermore, miR-143-3p inhibitor reversed the anti-proliferation and anti-metastasis effect of MEOX2-AS1 knockdown. Overall, the data indicated that the MEOX2-AS1/miR-143-3p/VDAC1 pathway participated in CC progression, making it a novel therapeutic target for CC cures.

## Graphical abstract

**Figure:**
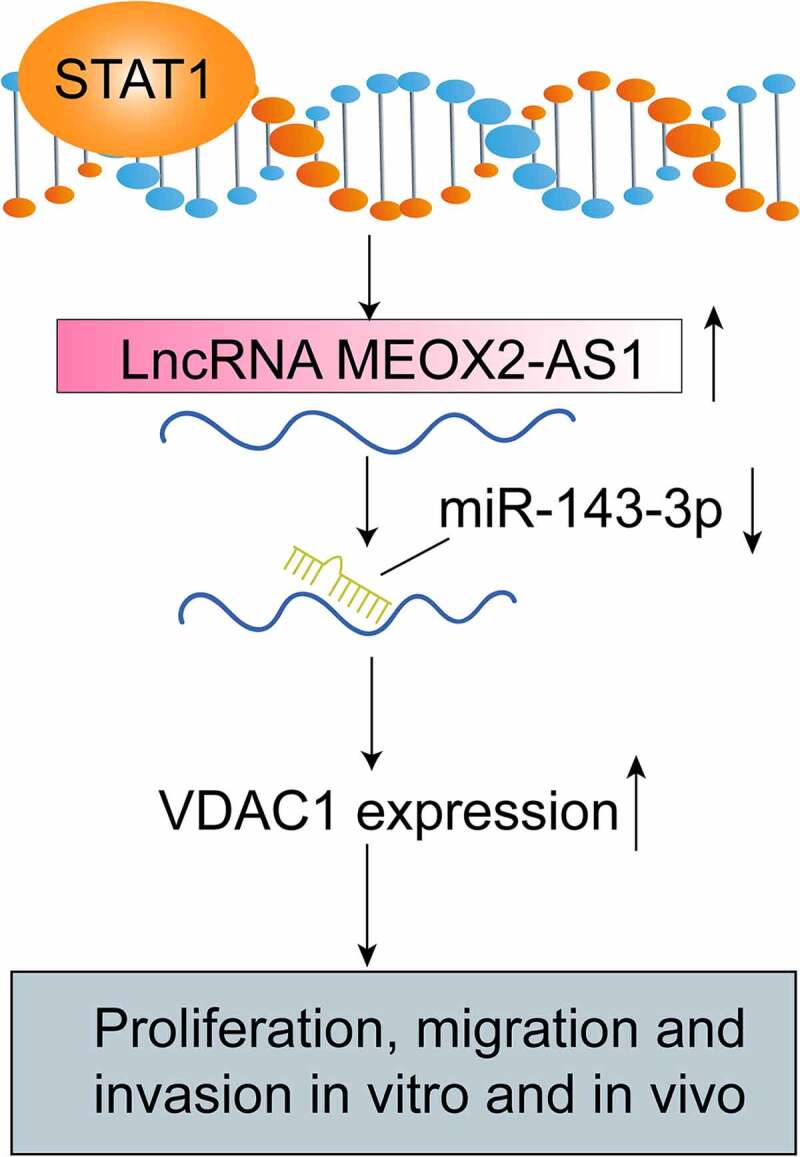

## Introduction

World-wide, cervical cancer (CC) is a prevailing malignancy as well as the second most frequent cause of tumor-induced deaths among women [1]. Approximately 520,000 new cases of CC were diagnosed each year according to the previous reports [2]. Genetic effects and viral infection have been demonstrated to be positively associated with this complex disease [3]. Despite the distinct progress in chemotherapy and surgical techniques, the great majority of patients with distant metastases exhibited an undesirable prognosis [4,5]. Indeed, the survival rate within five years of these patients is <12%. Thus, the determination of the underlying molecular mechanism in CC progression is helpful to find out new forceful therapeutic targets.

Long non-coding RNAs (lncRNAs) refer to RNA transcripts of > 200 nucleotides untranslated into protein [6]. They have attracted growing attention in the sector of life science. Over the past 20 years, numerous lncRNAs have been developed and named due to the developments of Chip sequencing and the abnormal expressions of lncRNAs have been implicated in several biological progresses [7,8]. More importantly, Abnormal expressions and functions of lncRNA are involved in the occurrence and developments of various diseases, especially malignant tumors, which indicated that lncRNAs could be utilized for tumor diagnosis and prognosis [9–11]. Therefore, it is essential to identify more tumor-associated lncRNAs and delve into their tumor-related functions and molecular mechanisms for the improvements of novel therapeutic methods of CC.

In recent years, the abnormal expressions of a novel lncRNA, MEOX2 antisense RNA 1(MEOX2-AS1), were found in several cancers including breast cancer and colon adenocarcinoma [12,13]. However, its specific function in tumors has not been researched. In this study, we aimed to explore the clinical significance and function of MEOX2-AS1 in CC. Here, the overexpression of MEOX2-AS1 in CC induced by STAT1 was evidenced. Then, we described the novel roles of miRNA-143-3p and MEOX2-AS1 in modulating voltage-dependent anion channel 1(VDAC1) to govern the metastatic progression of CC. As far as we know, this paper first directly reports MEOX2-AS1/miR-143-3p interaction as a crucial component in CC progression and metastasis through regulating VDAC1.

## Materials and methods

### Clinical samples

We retrospectively investigated 117 patients diagnosed with CC who underwent routine curative surgery between June 2015 and August 2017 at Dongying People’s Hospital. The patients had not received anti-tumor treatments before this study. Written informed consent from all patients and the approval by the Ethical Committee of Dongying People’s Hospital for all studies were obtained. Table 1 listed the clinicopathological characteristics.

**Table 1.** The primers used in this study for RT-PCR

Names	Sequences (5ʹ-3ʹ)
STAT1: F	CAGCTTGACTCAAAATTCCTGGA
STAT1: R	TGAAGATTACGCTTGCTTTTCCT
MEOX2-AS1: F	ACTGCTCTTGCATCCACTCAG
MEOX2-AS1: R	GGCACCTTCAACCTTCACTC
miR-143-3p: F	GGGGTGAGATGAAGCACTG
miR-143-3p: R	CAGTGCGTGTCGTGGAGT
VDAC1: F	ACGTATGCCGATCTTGGCAAA
VDAC1: R	TCAGGCCGTACTCAGTCCATC
GAPDH: F	GGAGCGAGATCCCTCCAAAAT
GAPDH: R	GGCTGTTGTCATACTTCTCATGG
U6: F	TGCGGGTGCTCGCTTCGGCAGC
U6: R	CCAGTGCAGGGTCCGAGGT

### Cell CULTURE AND TRANSFECTION

Normal cervical epithelial cells (H8) and human CC cell lines (including MS751, HeLa, C33A, and SiHa) were provided by Shanghai Institute of Cell Biology (Shanghai, China). RPMI 1640 medium supplemented with 100 mg/ml streptomycin, 100 U/ml penicillin, and 10% fetal bovine serum (Bioctyocare, Fanyu, Guangzhou, China) was used to culture cells in humidified air with 5% CO_2_ at 37°C.

Short hairpin RNA (shRNA) sequences targeting MEOX2-AS1 were designed. The shRNAs were inserted into lentiviral pHBLV/U6-Scramble-Luc-Puro^01^ vector (Zorin Technology, Suzhou, Jiangsu, China), named sh-MEOX2-AS1-1(AGTGGGAAACCCCACTATTTTCACACCT) and sh-MEOX2-AS1-2(CCACAAAGGTGCCTCAAGCATCCCACA); negative control was named sh-NC(CCACCGGTTAAGTCCTACTAC). Negative control siRNA (si-NC) and siRNA targeting STAT1(si-STAT1) were provided by T&L Biological Technology(Haidian, Beijing, China). MiRNA-143-3p mimics and miRNA-143-3p inhibitors together with their controls were provided by Ribo Co., Ltd. (Guangdong, China). Lipofectamine 2000 (Invitrogen, Hangzhou, Zhejiang, China) was applied to carried out plasmid transfections. The transfection efficiency of the above factors were demonstrated by the use of Western blot assays or RT-qPCR

### Bioinformatics analysis

The binding sites among MEOX2-AS1, miR-143-3p and VDAC1 were predicted using starBase (http://starbase.sysu.edu.cn/index.php). ‘GEPIA’(http://gepia.cancer-pku.cn/) analyzed the expression and clinical significance of STAT1 and VDAC1 in CC. The potential transcription factors were predicted via JASPAR online database(http://jaspar.genereg.net/).

### Quantitative real-time PCR (qRT-PCR)

TRIZOL reagent (Invitrogen, Haidian, Beijing, China) was applied to the extraction of total RNAs from cultured cells or frozen samples. Under the support of PrimeScript RT Reagent Kit (Bio-Rad, Hangzhou, Zhejiang, China), synthesis of complementary DNA (cDNA) was conducted. Next, on ABI 7500 using SYBR Premix ExTaq II kit (Takara, Hangzhou, Zhejiang, China), we performed qRT-PCR assays. The qRT-PCR data were analyzed, and calculated via the 2^−ΔΔCt^ methods. For normalization, GAPDH served as an endogenous control. Table 1 presented the used PCR primer sequences.

### Cell Counting Kit (CCK)-8 assays

The viability of CC cells was determined by CCK-8 assays (Liji Biology, Pudong, Shanghai, China). We seeded cells in 96-well microplates, changed the medium after treatments, and added CCK‐8 reagent (10 μL each well). By the use of a microplate reader, our group examined the absorbance at 450 nm.

### Clone formation

After seeding 1000 cells per well in 6-well plates, we cultured them in the full medium for 14 days, followed by a 30 min process of fixing cell colonies with 4% paraformaldehyde and a 30 min process of staining with 0.5% crystal violet.

### Wound healing assay

Wound healing assays were applied to the assessment of cell migration. The day before transfection, we seeded 3 × 10^5^ cells in 6 well plates and then used a 10-µl pipette to scratch the cells across the surface of the well. A medium containing 2% serum was used to incubate the wound cells for 24 h. At 0 and 24 h, an optical microscope (Olympus, Shenzhen, Guangdong, China) was used to photograph the images.

### Matrigel invasion assays

The invasion ability of CC cells for the exploration of MEOX2-AS1 function were evaluated by transwell assays. At 37°C, we used 25 mg Matrigel (BD Biosciences, Hangzhou, Zhejiang, China) to coat the upper filter membrane (pore diameter, 8 μm) of the Transwell plates for 30 min. The complete medium with 10% FBS was added in the lower chamber. We used serum-free DMEM to starve HeLa and SiHa cells for 4 h. After digestion, we transferred 1 × 10^5^ cells onto the upper surface and fixed the cells by paraformaldehyde at 4% in a way that the cells could penetrate to the lower surface for 15 min. Then, 4% paraformaldehyde was used to fix the migrated cells and crystal violet was used to stain them at room temperature for 30 min. Finally, the number of invaded cells was counted using a microscope.

### Animal study

Shanghai SIPPR-BK Laboratory Animal (Shanghai, China) provided BALB/c female nude mice which were used for in vivo experiments. We randomly classified mice with palpable tumors into two groups, six in each group. At a density of 5 × 10^6^, we injected HeLa cells stably transfected with sh-MEOX2-AS1-1 and sh-NC into nude mice. The detection of subcutaneous tumor volumes was performed by caliper every 4 days within 28 days and calculated as a × b^2^ × 0.5 (a, longest diameter; b, shortest diameter). Then, from killed mice, tumors were excised and weighed for further study. The Animal Protection Committee of Dongying People’s Hospital was followed throughout experiments under the approval of the Ethics Committee of Dongying People’s Hospital.

### Subcellular fractionation

The subcellular-related fractionating process for MEOX2-AS1 received the measuring process based on PARIS Tool (Life Technologies, Pudong, Shanghai, China).

### Luciferase reporter assays

In the promoter region of MEOX2-AS1, JASPAR was used to identify the STAT1 binding motif. After synthesis, we inserted fragment sequences into a pGL3-basic vector. Sequencing was conducted to verify all vectors and the Dual Luciferase Assay Kit (Promega) was applied for the detection of luciferase activities.

After using StarBase 2.0 and TargetScan 7.2 to predict binding sites between miR-143-3p and MEOX2-AS1 or VDAC1, we conducted site mutations, which were named VDAC1-mut and MEOX2-AS1-mut, respectively. VDAC1-mut and MEOX2-AS1-mut indicated that no site was bound to miR-143-3p, while VDAC1-wt and MEOX2-AS1-wt revealed some sites were bound to miR-143-3p. We then built pmirGLO-VDAC1-mut, pmirGLO-MEOX2-AS1-mut, PmirGLO-MEOX2-AS1-wt, and pmirGLO-VDAC1-wt vectors and cotransfected them into cells with NC mimics and miR-143-3p mimics. Then, 48 hours after transfection, we applied a dual luciferase reporter-gene kit (Promega) to examine luciferase activity.

### Western blots

The Total Protein Extraction kit was applied to the extraction of total protein. SDS-PAGE electrophoresis was used to separate identical quantities of proteins, which were then transferred onto nitrocellulose filter membranes. Primary antibodies specific for STAT1, N-cadherin, Vimentin, E-cadherin, VDAC1 or GAPDH were used to incubate the blots that were blocked with bovine serum albumin overnight at 4°C. GAPDH served as an endogenous control. At room temperature, we used goat anti-rabbit IgG secondary antibody conjugated to horseradish peroxidase (1:5000, abcam) to subsequently incubate the membranes for 2 h. All antibodies were purchased from Aviva Technology(Haidian, Beijing, China). An Imaging System was used to scan the bands.

### Statistical analysis

SPSS 19.0 software (SPSS, Armonk, NY, USA) was applied to data analysis, where mean ± standard error of the mean (SD) was used to express the data. Wilcoxon test, χ^2^ test or Student’s t-test was performed to determine the significance of differences between groups. The survival curve was calculated through the Kaplan-Meier method. In the multivariate assays, independent prognostic factors were assessed. Statistical significance was defined as a p-value of less than 0.05.

## Results

### MEOX2-AS1 is strongly upregulated in CC

**Figure 1.**
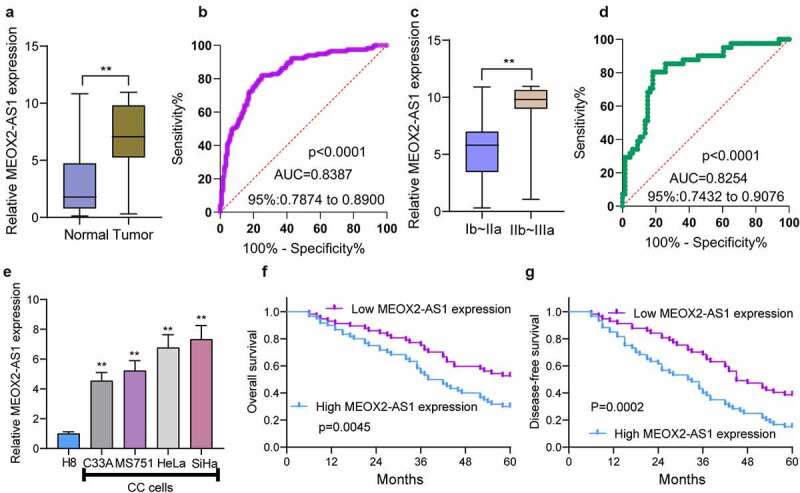
The distinct upregulation of MEOX2-AS1 in CC and its clinical significance. (a) The levels of MEOX2-AS1 in 117 patients using RT-PCR. (b) The diagnostic value of MEOX2-AS1 expressions was determined by ROC assays. (c) The comparison of MEOX2-AS1 levels between CC specimens with different stages. (d) The diagnostic value of MEOX2-AS1 for advanced CC specimens. (e)In different CC cell lines, MEOX2-AS1 levels were examined. (f, g) Kaplan-Meier curves for OS and DFS in 117 patients with CC divided based on MEOX2-AS1 expressions. **P < 0.01

To find out the abnormal expression of MEOX2-AS1 in CC, we performed qRT-PCR in the specimens of 117 CC patients, finding higher MEOX2-AS1 expression levels in CC specimens than those in matched non-tumor tissues (Figure 1(a)). An AUC value of 0.8387 (95% CI: 0.7874 to 0.8900) for CC was obtained by high MEOX2-AS1 expression, as revealed by ROC assays (Figure 1(b)). Also, compared with those with the early stage, we observed a higher level of MEOX2-AS1 from CC specimens with advanced stages (Figure 1(c)). High MEOX2-AS1 expression presented diagnostic value in distinguishing CC specimens of advanced stages from specimens of early stages as demonstrated by an AUC value of 0.8254(95% CI: 0.7432 to 09076)(Figure 1(d)). Furthermore, the normal cervical epithelial cells (H8) had increased MEOX2-AS1 expressions compared with CC cell lines SiHa, HeLa, MS751 and C33A (Figure 1(e)).

### Prognostic values of MEOX2-AS1 levels in CC

To explore the clinical significance of MEOX2-AS1 expressions in CC, according to the median expressions of MEOX2-AS1 (6.73) in all CC samples, our group divided the 117 CC patients into two groups, low-expression (n = 57) and high-expression (n = 60). The results of chi-square test indicated the positive correlation of high MEOX2-AS1 expression with depth of cervical invasion(p = 0.017), lymph node metastasis(p = 0.012) and FIGO stage(p = 0.007) (Table 2). Compared with the low expression group, the high expression group presented shorter disease-free survival(DFS) (p = 0.0002, Figure 1(g)) and overall survival(OS)(p = 0.0045, Figure 1(f)), as revealed by the Kaplan-Meier survival analysis. More importantly, MEOX2-AS1 expression was confirmed by Multivariate analysis to be an independent prognostic factor for both 5-year OS(HR = 2.933, 95% CI: 1.329–4.673, p = 0.006) and 5-year DFS(HR = 3.132, 95% CI: 1.344–5.132, p = 0.003) in CC(Table 3).

**Table 2.** Clinicopathological features associated with MEOX2-AS1 expression in 117 CC patients

		MEOX2-AS1 expression		P value
Clinicopathological features	Total	High	Low	
Age				0.499
<45	64	31	33	
≥45	53	29	24	
Tumor size (cm)				0.228
<4.0	59	27	32	
≥4.0	58	33	25	
FIGO stage				0.007
Ib~IIa	76	32	44	
IIb~IIIa	41	28	13	
Lymph node metastasis				0.012
No	84	37	47	
Yes	33	23	10	
Depth of cervical invasion				0.017
<2/3	80	35	45	
≥2/3	37	25	12

**Table 3.** Multivariate analysis of overall survival and disease-free survival in 117 patients with CC

Risk factors	Overall survival			Disease-free survival		
	HR	95% CI	*p*	HR	95% CI	*p*
Age	0.873	0.432–1.467	0.351	0.954	0.556–1.732	0.411
Tumor size	1.013	0.544–1.893	0.432	1.211	0.654–2.033	0.324
FIGO stage	3.113	1.341–5.456	0.003	3.432	1.544–6.432	0.001
Lymph node metastasis	2.893	1.266–4.673	0.006	3.231	1.332–6.763	0.001
Depth of cervical invasion	2.678	1.241–4.545	0.013	2.745	1.327–4.993	0.008
MEOX2-AS1 expression	2.933	1.329–4.673	0.006	3.132	1.344–5.132	0.003

### STAT1 activated MEOX2-AS1 expressions in CC

**Figure 2.**
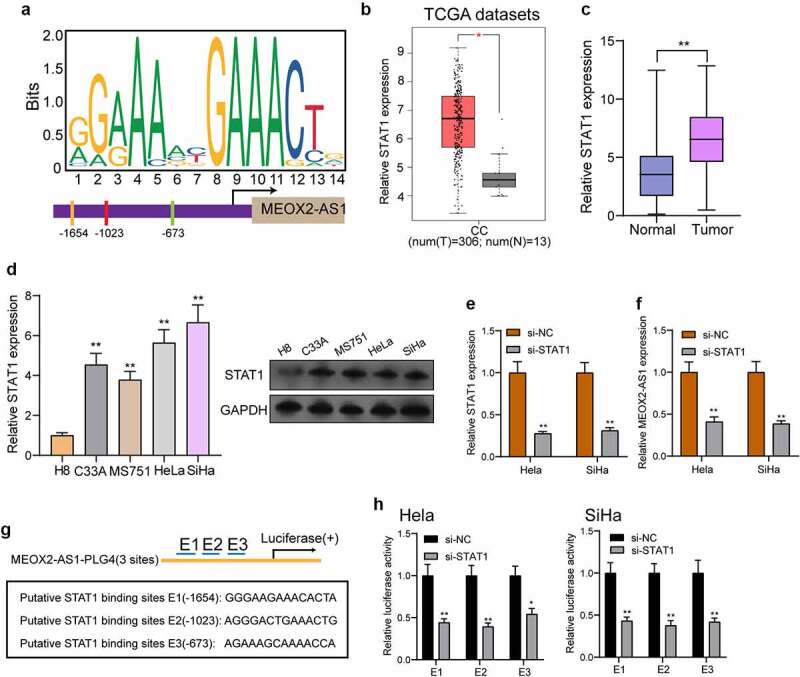
STAT1 activates the transcription of MEOX2-AS1. (a) JASPAR predicted STAT1 binding site prediction in the MEOX2-AS1. we obtained DNA motif of STAT1 and predicted three binding sites of STAT1 in MEOX2-AS1 promoter. (b) The STAT1 expressions in 306 CC specimens and 13 non-tumor cervical specimens from TCGA datasets. (c) The expressions of STAT1 in our cohort. (d) RT-PCR determined STAT1 expressions in different CC cell lines. (e, f) STAT1 and MEOX2-AS1 expressions in CC cells transfected with si-STAT1 or si-NC. (g) Construction of the luciferase reporter vector. (h) Luciferase activity was distinctly decreased in si-STAT1-transfected cells compared with control vector in three binding sites. **P < 0.01, *p < 0.05

To explore the potential mechanisms involved in MEOX2-AS1 dysregulation, we focused on transcription factors which has been reported to exhibit he regulatory effects on the expressions of lncRNAs [14,15]. After searching the JASPAR online database, our attention focused on STAT1 which exhibited high scores, for the follow-up study. Figure 2(a) presents the predicted binding sites of STAT1 in the MEOX2-AS1 promoter sequence. Then, we analyzed TCGA datasets and found that the levels of MEOX2-AS1 in CC specimens were higher than those in normal cervical tissues(Figure 2(b)), which was also demonstrated in our cohort and CC cell lines using RT-PCR and Western blot (Figure 2(c,d)). Moreover, MEOX2-AS1 expressions were distinctly hindered in Hela and Siha cells after knockdown of STAT1 (Figure 2(e,f)). Besides, a PGL4 luciferase reporter vector was inserted by 3 potential binding sites of STAT1 and the MEOX2-AS1 promoter region (Figure 2(g)). Furthermore, the results of dual-luciferase reporter assays revealed that knockdown of STAT1 could inhibit the luciferase activity (Figure 2(h)). Our findings suggested that the up-regulation of MEOX2-AS1 in CC cells may be induced by STAT1.

### Silencing MEOX2-AS1 repressed CC cell proliferation and metastasis

**Figure 3.**
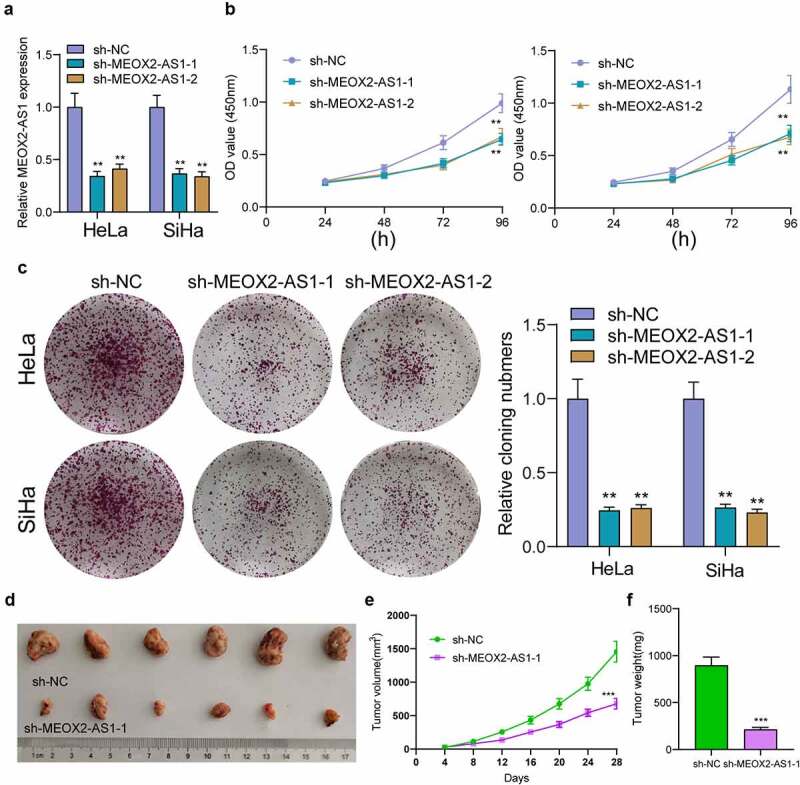
**Silence of MEOX2-AS1 suppressed the proliferation of CC cells**. (a) MEOX2-AS1 expressions in CC cells, transfected with sh-MEOX2-AS1-1, sh-MEOX2-AS1-2 or sh-NC, were tested by qRT-PCR. (b) The proliferative abilities of Hela and SiHa cells were determined through CCK8 assays. (c) Colony formation assays. (d) Tumors from sh-MEOX2-AS1 group and sh-NC group were displayed. (e,f)Volume and weight of tumors obtained were shown. **p < 0.01, ***p < 0.001

**Figure 4.**
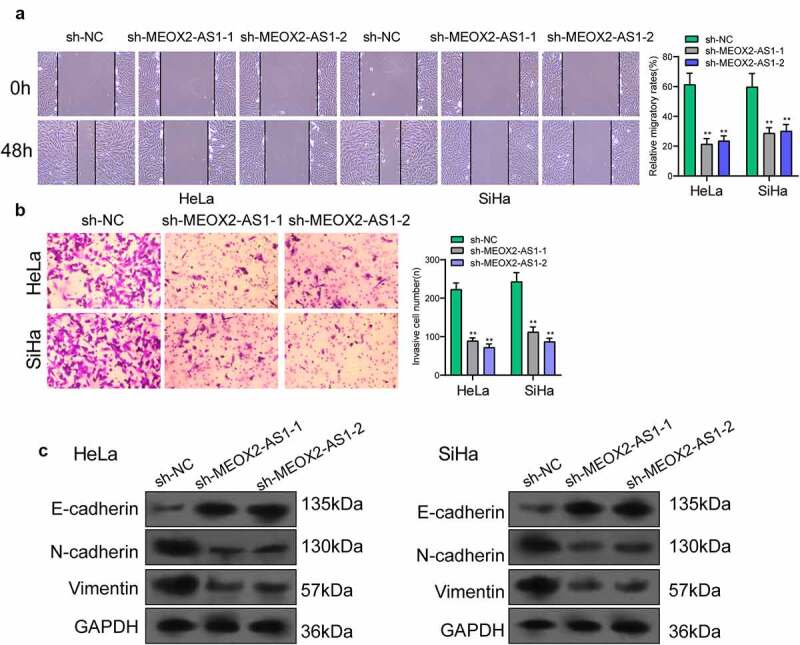
**Knockdown of MEOX2-AS1 decreases the invasion and migration potential of Hela and SiHa cells**. (a) representative images and quantitative analysis of scratch assays, delayed closure was observed in CC cells with MEOX2-AS1 knockdown. (b) Transwell assays demonstrated the anti-invasive ability of sh- MEOX2-AS1-1 and sh-MEOX2-AS1-2. (c) Western blot assays of EMT pathways. **p < 0.01

The MEOX2-AS1 loss-of-function experiments in SiHa and HeLa cells were applied to study the effect of MEOX2-AS1 on CC cells. After transfection with sh-MEOX2-AS1-1 or sh-MEOX2-AS1-2, the expressions of MEOX2-AS1 was significantly declined in CC cells (Figure 2(a)). After CC cells were transfected with MEOX2-AS1 shRNA, both cell proliferation in CCK-8 assays (Figure 3(b)) and colony formation in colony formation assays (Figure 3(c)) were reduced. Compared with the control group, nude mice after subcutaneous injection with sh-MEOX2-AS1-1 presented slower tumor growth, as revealed by vivo assays (Figure 3(d)). Moreover, compared with control group, the tumor volume and weight were apparently lessened in sh-MEOX2-AS1-1 group (Figure 3(e,f)). To study whether silence of MEOX2-AS1 exhibited a regulatory effect on CC cells’ metastasis, wound healing assay was performed, which showed the inhibited migration ability of CC cells after MEOX2-AS1 knockdown (Figure 4(a)). Moreover, transwell assays also revealed a distinct decrease in the invasion ability of SiHa and HeLa cells transfected with sh-MEOX2-AS1-1 and sh-MEOX2-AS1-2 (Figure 4(b)). Tumor cells acquire mesenchymal phenotype and metastasize toward distant sites via EMT progress. Then, based on the results of Western blot in EMT markers, we observed the lower expressions of N-cadherin and vimentin and higher expressions of E-cadherin in CC cells after knockdown of MEOX2-AS1 (Figure 4(c)).

### MEOX2-AS1 acted as a miR-143-3p sponge in CC cells

**Figure 5.**
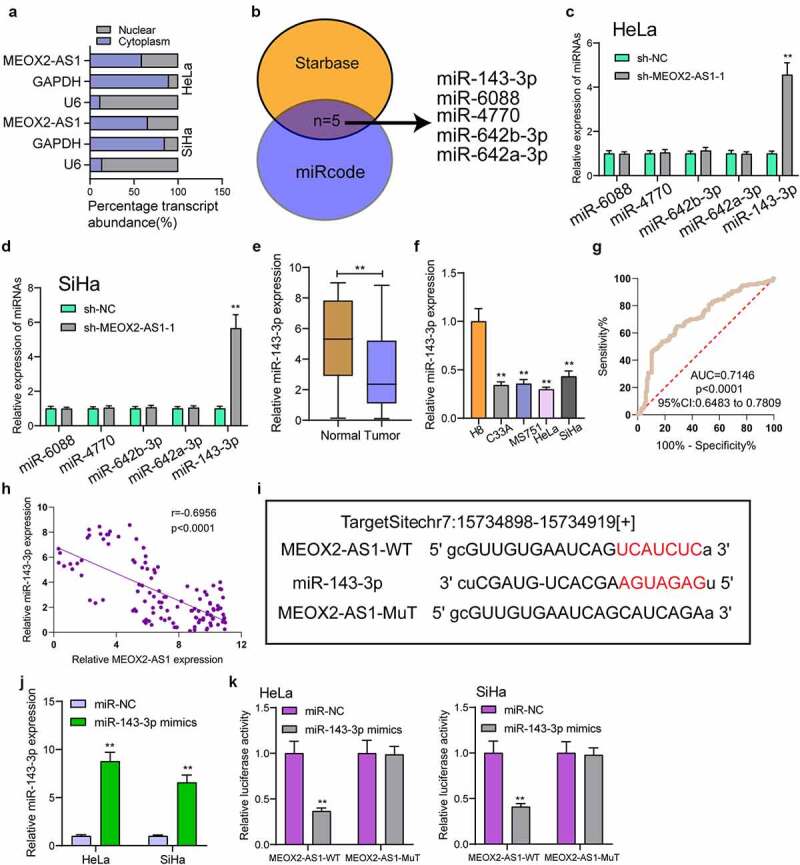
**MEOX2-AS1 acts as a sponge for miR-143-3p**. (a) Subcellular fractionation assays for the location of MEOX2-AS1 in SiHa and Hela cells. (b) The prediction of the putative miRNAs targeting MEOX2-AS1 using miRcode and StarBase 3.0. (c,d)RT-PCR for the detection of the miRNA levels in MEOX2-AS1-depleted Hela and SiHa cells. (e) Decreased levels of miRNA-143-3p were observed in CC. (f) RT-PCR confirmed decreased expression of miR-143-3p in four CC cells compared to H8 cells. (g) The diagnostic value of miRNA-143-3p expressions in our cohort. (h) Pearson’s correlation coefficient showed the relationship between miR-143-3p and MEOX2-AS1 in the 117 CC tissues. (i) Schematic outlining the predicted binding sites between MEOX2-AS1 and miR-143-3p. (j) MiR-143-3p mimics promoted the expression of miR-143-3p in Hela and SiHa cells. (k) MiR-143-3p mimics obviously reduced the luciferase activity of MEOX2-AS1-WT. **p < 0.01

For exploring the underlying mechanisms involved in MEOX2-AS1 function in CC, we performed subcellular fractionation, finding high expression of MEOX2-AS1 in the cytoplasm of HeLa and SiHa cells (Figure 5(a)). Five miRNAs (miR-642a-3p, miR-642b-3p, miR-4770, miR-6088 and miR-143-3p) had complementary sequence pairing with MEOX2-AS1 (Figure 5(b)), according to online databases (miRcode and StarBase version 3.0). MEOX2-AS1’s ability to modulate these candidates’ expression in CC cells was examined by RT-PCR. We observed that with the down-regulation of MEOX2-AS1, miRNA-143-3p expressions in HeLa and SiHa cells increased distinctly, while no change was found in expressions of the other miRNAs (Figure 5(c,d)). Moreover, the down-regulation of miRNA-143-3p in cells and 117 CC specimens was found (Figure 5(e,f)). The diagnostic value of miRNA-143-3p was also demonstrated in our cohort (Figure 5(g)). A negative correlation of miRNA-143-3p expression with MEOX2-AS1 expressions was revealed by correlation analysis (Figure 5(h); r = −0.6956, p < 0.0001). MiRNA-143-3p played a significant role in reducing the relative luciferase activity of the wild-type MEOX2-AS1, as found from the dual-luciferase reporter assay (Figure 5(i-k)). Our findings suggested that MEOX2-AS1 directly ‘sponges’ miRNA-143-3p.

### MEOX2-AS1 knockdown suppresses VDAC1 expression via miR-143-3p

**Figure 6.**
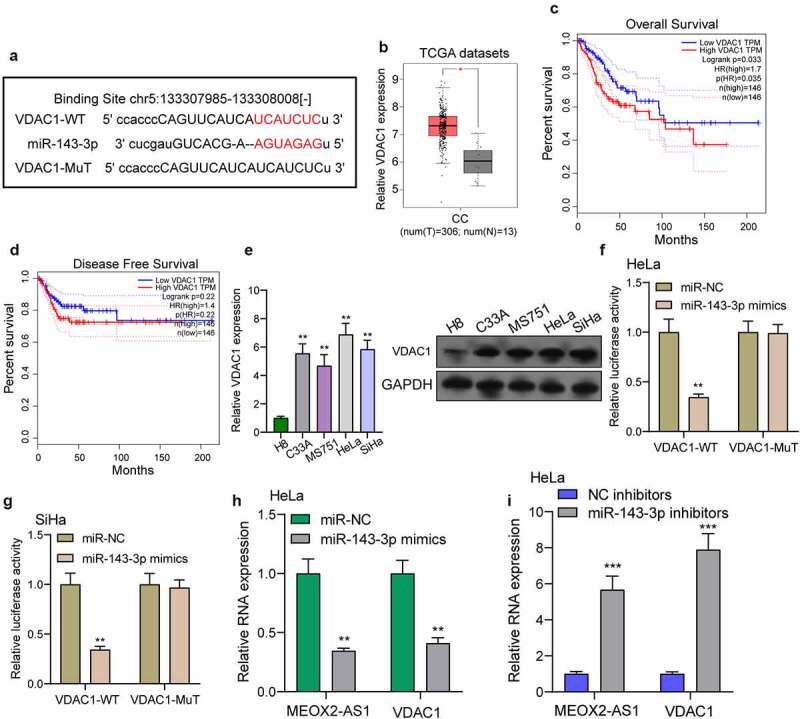
**VDAC1 was a direct target of miR-143-3p**. (a) Schematic construction of WT and Mut 3ʹ-UTR of VDAC1 were displayed. (b) VDAC1 expression in 306 CC tissues and 13 non-tumor samples(TCGA datasets). (c,d) Kaplan-Meier curves for OS and DFS in 292 patients with CC. (e) The expression of VDAC1 in different CC cells. (f,g) Dual luciferase reporter assays demonstrated the functions of miR-143-3p mimics on the activity of the 3ʹUTRs of VDAC1 in Hela and SiHa cells. (h) The levels of MEOX2-AS1 and VDAC1 in Hela cells transfected with miR-NC or miR-143-3p mimics. (i) The levels of MEOX2-AS1 and VDAC1 in Hela cells transfected with NC inhibitors or miR-143-3p inhibitors. ***p < 0.001 **p < 0.01, *p < 0.05

**Figure 7.**
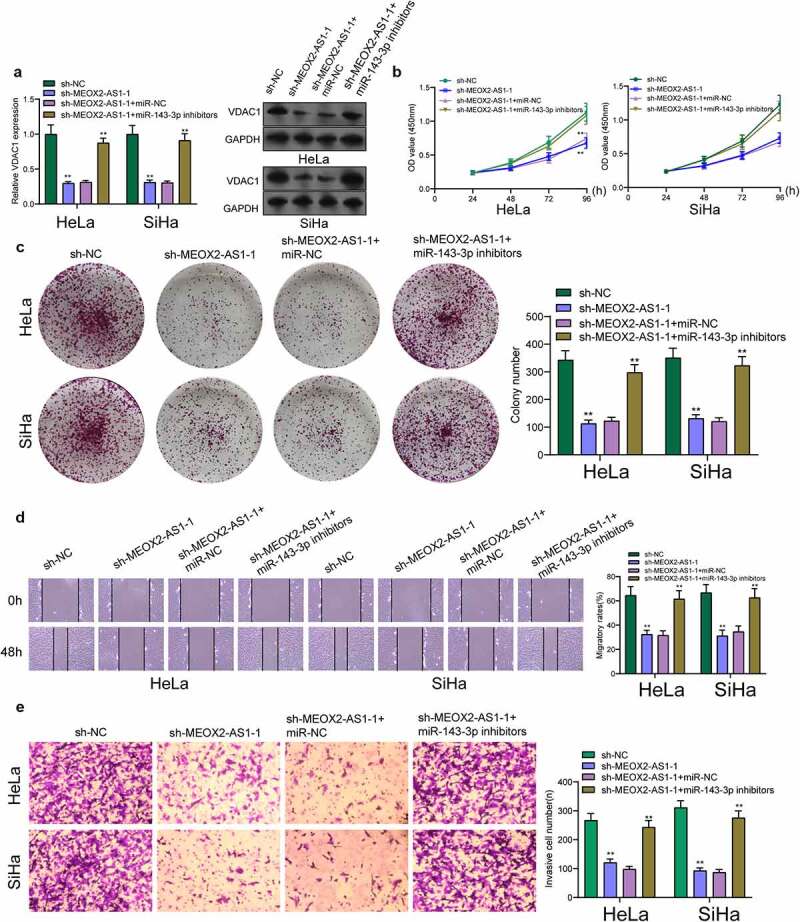
**Knockdown of miR-143-3p attenuates the regulatory functions of MEOX2-AS1 silence on the progression of CC cells**. (a) The expression levels of VDAC1 in SiHa and Hela cells after knockdown of MEOX2-AS1 and/or inhibition of miR-143-3p. The CCK-8 assays (b), colony formation assays (c), Cell migration(d) and cell invasion assays (e) following knockdown of MEOX2-AS1 and/or inhibition of miR-143-3p. *p < 0.05, **p < 0.01

We further explored the underlying molecular mechanisms of MEOX2-AS1/miR-143-3p axis in regulating CC development. According to the prediction of TargetScan software, miR-143-3p target sites were found in the 3ʹUTR of VDAC1 mRNA (Figure 6(a)). By analyzing TCGA datasets, we observed that VDAC1 was overexpressed in CC specimens compared with non-tumor cervical specimens (Figure 6(b)). In addition, survival assays using TCGA datasets suggested that patients with high VDAC1 exhibited a shorter OS (Figure 6(c)). However, the distinct association was not observed between patients with VDAC1 expression and DFS (Figure 6(d)). Moreover, VDAC1 expression at protein and mRNA levels was distinctly increased in four CC cells (Figure 6(e)). Compared to cells transfected with the control mimic, overexpression of miRNA-143-3p induced approximately 61% reduction in luciferase reporter activity in Hela cells and 64% reduction in Siha cells, while miR-143-3p did not affect the mutated reporter activity (Figure 6(f,g)), as found from luciferase reporter assays. Furthermore, RT-PCR revealed increased expressions of MEOX2-AS1 and VDAC1 in Hela cells after the transfection of miRNA-143-3p mimics, but decreased expressions after knockdown of miR-143-3p (Figure 6(h,i)). To further explore whether MEOX2-AS1 regulated CC progression by adjusting miRNA-143-3p/VDAC1 axis, we performed rescue experiments and found that with the downregulation of miRNA-143-3p, the role of MEOX2-AS1 knockdown in hindering the expression of VDAC1 was weakened (Figure 7(a)). Moreover, the results of a series of functional assays revealed that downregulation of miRNA-143-3p weakened the suppressive roles of MEOX2-AS1 knockdown in the invasion, migration and proliferation of SiHa and HeLa cells (Figure 7(b-e)).

## Discussion

Over the past decades, many studies showed that some functional lncRNAs were dysregulated in various types of tumors [16,17]. Their roles in regulating tumor proliferation and metastasis suggested them as novel therapeutic targets and biomarkers [18,19]. Previously, the correlation of several lncRNAs including lncRNA TPT1-AS1 and lncRNA HAND2-AS1 with the clinical outcome of CC patients has been reported [20,21]. Here, a novel CC-related lncRNA, MEOX2-AS1, was identified, which was overexpressed in both cell lines and CC specimens. Previously, high MEOX2-AS1 expression was also observed in colon adenocarcinoma and breast cancer [12,13]. In our cohort, we also demonstrated its diagnostic value in distinguishing CC specimens from normal cervical specimens. The correlation of high MEOX2-AS1 expression with poor prognosis and advanced clinical progress was found by clinical assays. Our findings suggested that MEOX2-AS1 was a novel prognostic and diagnostic biomarker for CC patients.

Up to date, although many lncRNAs have been demonstrated to display a dysregulated level in tumors, the potential mechanisms involved in their dysregulation remained largely unclear [22,23]. In recent years, it was found from some research that some transcript factors (TF) could regulate lncRNA expression, including SP1 regulating lncRNA SPRY4-IT1 expression and E2F1 regulating lncRNA LMCD1-AS1 [24,25]. In this study, we showed high MEOX2-AS1 was induced by STAT1. STAT1 was a transcript factor promoting CC cells’ proliferation and metastasis. In addition, several lncRNAs were reported to be induced by STAT1, such as lncRNA KTN1-AS1 in lung cancer and lncRNA LINC00467 in lung adenocarcinoma [26,27]. We also observed STAT1 was highly expressed in CC specimens from our cohort. The involvement of lncRNAs in the biological function of cancer cells has been reported. It was also found that after the silence of MEOX2-AS1, CC cells’ proliferation, metastasis and EMT progress were hindered. These data suggested that STAT1-induced upregulation of MEOX2-AS1 promoted the progression of CC.

Based on substantial data, lncRNA transcripts can be considered endogenous decoys for miRNAs, which, via their miRNA binding sites, affect cancer-related gene expression [28]. For instance, overexpression of Linc00887 weakened CC cells’ metastasis ability by regulating the miRNA-454-3p/FRMD6-Hippo axis [29]. High expression of lncRNA DLX6-AS1 was observed in CC. Moreover, its knockdown modulated miRNA-16-5p/ARPP19 to inhibit the proliferation and invasion of CC cells [30]. It has been confirmed that many cytoplasmic lncRNAs as competing endogenous RNAs (ceRNAs) competitively bind microRNAs [31,32]. It was found that the cytoplasm displayed a larger proportion of MEOX2-AS1 expression. Moreover, we found that MEOX2-AS1 could directly bind miRNA-143-3p as well as down-regulated miRNA-143-3p expression in CC cells. Previously, low expressions of miRNA-143-3p were found in some tumors, including CC, while its overexpression could inhibit CC cells’ proliferation and metastasis [33,34]. Thus, these findings indicated that MEOX2-AS1 may serve as an oncogenic lncRNA through sponging miR-143-3p.

The role of miRNAs in regulating gene expressions on the post-transcriptional levels has been demonstrated. It achieves this goal through binding to the 3ʹ-UTR of the target mRNAs, resulting in translational or degradation repression [35]. Previously, miR-143-3p displayed regulatory functions in tumor progression via targeting several mRNAs [33,34]. Here, bioinformatics analysis revealed VDAC1 as a potential target of miRNA-143-3p. This result was further demonstrated by Luciferase activity assays. Previously, VDAC1 was reported to promote CC cells’ invasion and proliferation. It was observed as well that VDAC1 expression was distinctly increased in CC, which was in line with previous findings [36,37]. Then, we wondered whether MEOX2-AS1 facilitated CC progression by miRNA-143-3p/VDAC1. The results of rescue experiments demonstrated that after the silence of miR-143-3p, the anti-oncogenic roles of MEOX2-AS1 knockdown in invasion, migration and proliferation were weakened. Overall, the above findings implied the role of STAT1/MEOX2-AS1/miR-143-3p/VDAC1 axis in regulating CC cells’ metastasis, proliferation, and EMT progress.

## Conclusion

We first provided evidence that an overexpressed lncRNA in CC, MEOX2-AS1, facilitated CC cells’ metastasis and proliferation. We suggested that STAT1-induced MEOX2-AS1 as a sponge of miRNA-143-3p activates CC carcinogenesis by positively regulating VDAC1 expression. The research results revealed MEOX2-AS1 to be a novel prognostic biomarker in CC detection and a promising therapeutic target for CC treatments.

## Data Availability

All data that support the findings of this study are available from the corresponding authors upon reasonable request.
